# Extracellular vesicles isolated from porcine seminal plasma exhibit different tetraspanin expression profiles

**DOI:** 10.1038/s41598-019-48095-3

**Published:** 2019-08-09

**Authors:** Isabel Barranco, Lorena Padilla, Inmaculada Parrilla, Alberto Álvarez-Barrientos, Cristina Pérez-Patiño, Fernando J. Peña, Emilio A. Martínez, Heriberto Rodriguez-Martínez, Jordi Roca

**Affiliations:** 1 Department of Medicine and Animal Surgery, Faculty of Veterinary Science, University of Murcia, Murcia, 30100 Spain; 2 Applied Bioscience Facility, University of Extremadura, Badajoz, 06006 Spain; 3 Laboratory of Equine Reproduction and Equine Spermatology, University of Extremadura, Caceres, 10003 Spain; 4 Department of Clinical and Experimental Medicine (IKE), University of Linköping, Linköping, SE 58185 Sweden

**Keywords:** Reproductive biology, Transmission electron microscopy

## Abstract

Seminal extracellular vesicles (EVs) include exosomes (ø 40–120 nm) and microvesicles (MVs, ø 120–1000 nm), which would be involved in multiple functional reproductive roles. The study aimed to establish which EV subtypes are present in pig semen, using a high-resolution flow cytometer to explore differences in their tetraspanin expression profile. The EVs were isolated from 12 pig ejaculates using serial ultracentrifugation and characterized by dynamic light scattering and electron microscopy for size and morphology as well as for tetraspanin expression using flow cytometry with Carboxyfluorescein succinimidyl ester (CFSE) and antibodies against CD9, CD63 and CD81. Pig semen contained a heterogeneous EV-population regarding size and morphology. Flow cytometric analysis demonstrated that the proportion of EVs expressing CD63 and CD9 was higher in MVs (*P* < 0.001 and *P* < 0.05, respectively) than in exosomes, while the opposite was true for CD81; higher (*P* < 0.001) in exosomes than in MVs. In conclusion, (1) the new generation of flow cytometers are able to accurately identify EVs and to gate them in two size-different populations named exosomes and MVs. (2) Tetraspanins CD9, CD63 and CD81 are present in both seminal EVs, albeit with exosomes and MVs differing in expression profiles, suggesting dissimilar cargo and binding affinity.

## Introduction

Over the last years, the involvement of the cargo of mammalian extracellular vesicles (EVs), as exosomes or microvesicles (MVs), for autocrine and paracrine signaling during physiological and pathological processes has gained the interest of the scientific community^1^. Consequently, the identification of EVs as potential non-invasive biomarkers of pathological processes or their use as possible therapeutic vehicles is frontline research in biomedicine^2–5^. The EVs are widely distributed throughout the mammalian body, being isolable from most body fluids^6–8^, including semen^9–12^. Although the potential role of seminal plasma EVs in reproductive function has not been fully defined, they seem to play key signaling during sperm maturation, motility and capacitation^9,13^. In pigs, seminal exosomes are specifically involved in sperm capacitation, sperm-zona pellucida binding^14,15^ and modulation of endometrial immune-related gene expression^12^.

Exosomes and MVs differ in size, intracellular origin and shedding to the extracellular milieu. Exosomes are small EVs, ranging between 40 to 120 nm in diameter (ø), and are released when multivesicular endosomes or multivesicular bodies fuse with the plasma membrane^16^. The MVs are larger in size, 120 to 1000 nm of ø, directly shed by the plasma membrane^17^. Despite extensive research on EVs, no technique has been established to separate different EV subtypes. This hampers our capability to identify the specific cargo and function of each of the EV subtypes. Therefore, studies addressing this issue are of outmost importance.

A new generation of flow cytometers together with the use of primary antibodies against tetraspanins provide a useful tool to characterize EVs^18,19^. Tetraspanins are highly conserved plasma membrane proteins that act as scaffolds for other proteins in particular areas of the cell plasmalemma^20^. Owing to their endocytic or plasma membrane origin, EVs present particular tetraspanins, which helps to identify the EVs but also to define their cargo and/or their target cells^21,22^. The most conspicuous tetraspanins in EVs are CD9, CD63 and CD81; the latter two classically used as specific exosome markers^22^. However, they are reported as also present in MVs^7^, jeopardizing their use as specific exosome markers. Lötvall *et al*.^23^ and Colombo *et al*.^1^ suggested that differences in tetraspanin expression, as opposed to their mere presence, between both EV subtypes could alleviate the confounding findings. A quantitative analysis of tetraspanins should thus be performed to differentiate between these EV subtypes.

Therefore, the present study aimed quantitation of the expression of CD9, CD63 and CD81 tetraspanins in EVs present in pig seminal plasma using novel high-resolution flow cytometry. The EVs were isolated by serial ultracentrifugation and characterized by dynamic light scattering (DLS) for size distribution and transmission (TEM) and scanning (SEM) electron microscopy for ultrastructural aspect and size measuring. In addition to the relevance for pig reproduction, the expected results would be interesting for other mammalian species, including human, considering pig is a useful animal model for biomedical research^24^.

## Results

### Ultrastructure and size distribution of EVs

**Figure 1:**
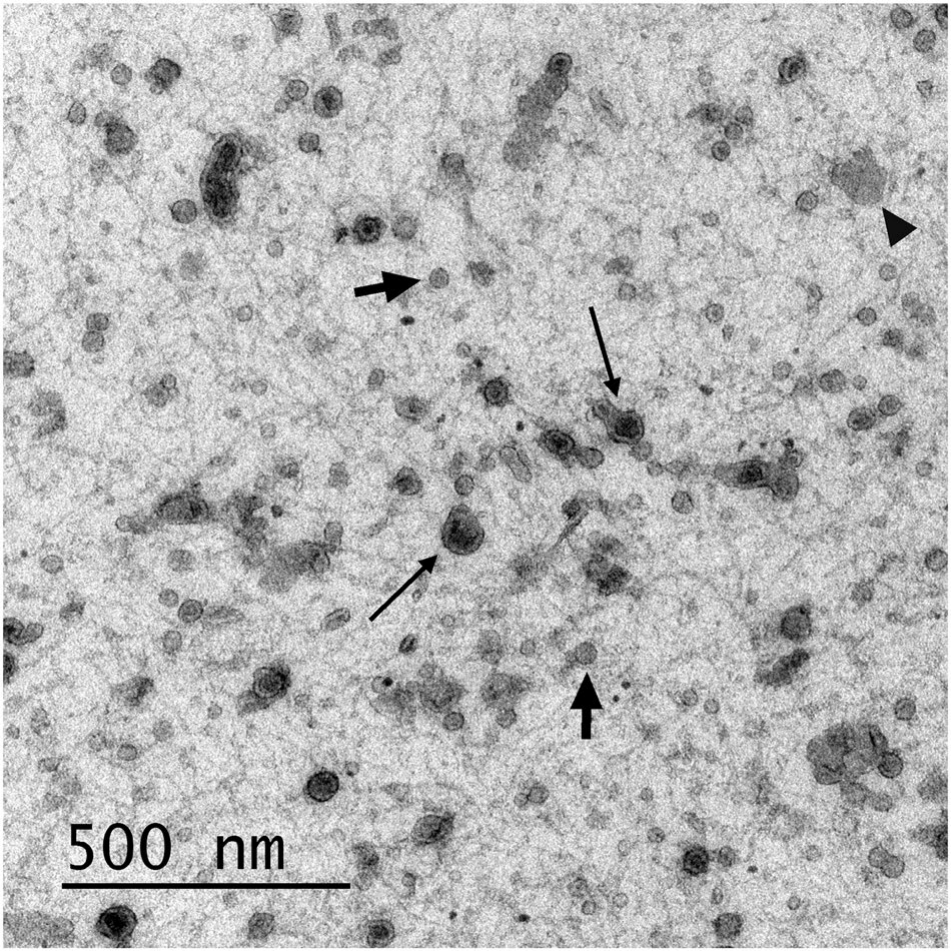
Transmission electron micrograph of extracellular vesicles isolated from pig seminal plasma. The extracellular vesicles were isolated by serial ultracentrifugation and contrasted in 2% uranyl-oxalate and embedded in a mixture of 4% uranyl-acetate. Larger vesicles (microvesicles) enclosed a granule electron-dense material (thick arrows) and the smaller ones (exosomes) showed a less dense inner content (short arrows). Amorphous material (arrow heads) in the background was compatible with protein aggregates.

**Figure 2:**
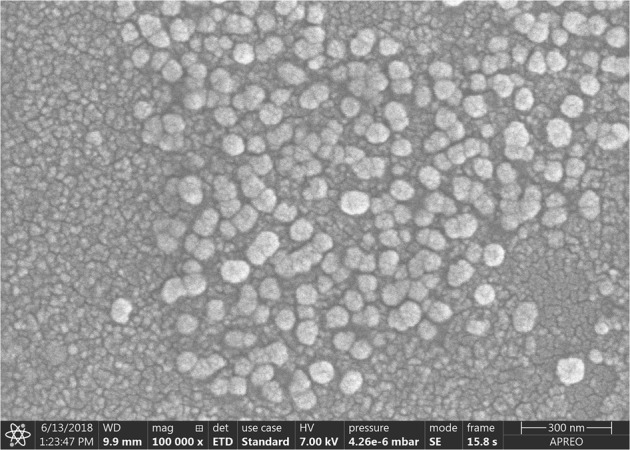
Scanning electron micrograph of extracellular vesicles isolated from pig seminal plasma by serial ultracentrifugation.

Electron microscopic imaging confirmed that pig semen contains EVs (Figs 1 and 2). The EVs-preparations from different ejaculates (n = 12) displayed similar characteristics. Images acquired by TEM revealed the presence of a heterogeneous population of vesicles of different sizes (Fig. 1). All EVs showed round or cup-shaped morphology and were surrounded by a single or double membrane. The less frequent larger vesicles (MVs), enclosed a granule electron-dense material, while the smaller ones (in majority, exosomes) had a less dense inner content. Some TEM images displayed an amorphous material in the background, compatible with protein aggregates (Fig. 1). Other electron-dense particles were not observed outside the vesicles. The SEM images confirmed the presence of vesicles with spherical morphology and different size in the EVs-preparations (Fig. 2).

**Figure 3:**
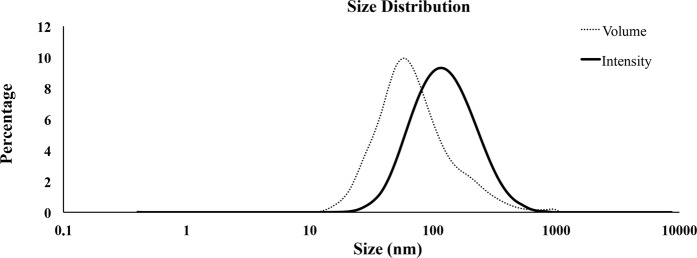
Dynamic Light Scattering (DLS) measurements. Particle size distribution of extracellular vesicles isolated from pig seminal plasma by serial ultracentrifugation, assessed by DLS using Nano Zetasizer. Each curve represents an average of volume (dotted line) and intensity (solid line) size distributions of all semen samples (n = 12).

The DLS analysis evidenced that EVs present in the seminal plasma exhibited an asymmetric size distribution. The EVs diameter ranged from 13.55 to 824.99 nm and 77% of all vesicles had a diameter between 32.67 to 122.41 nm (Fig. 3). The EVs size distribution pattern remained similar among ejaculates (n = 12).

### Tetraspanin expression of porcine seminal plasma EVs

**Figure 4:**
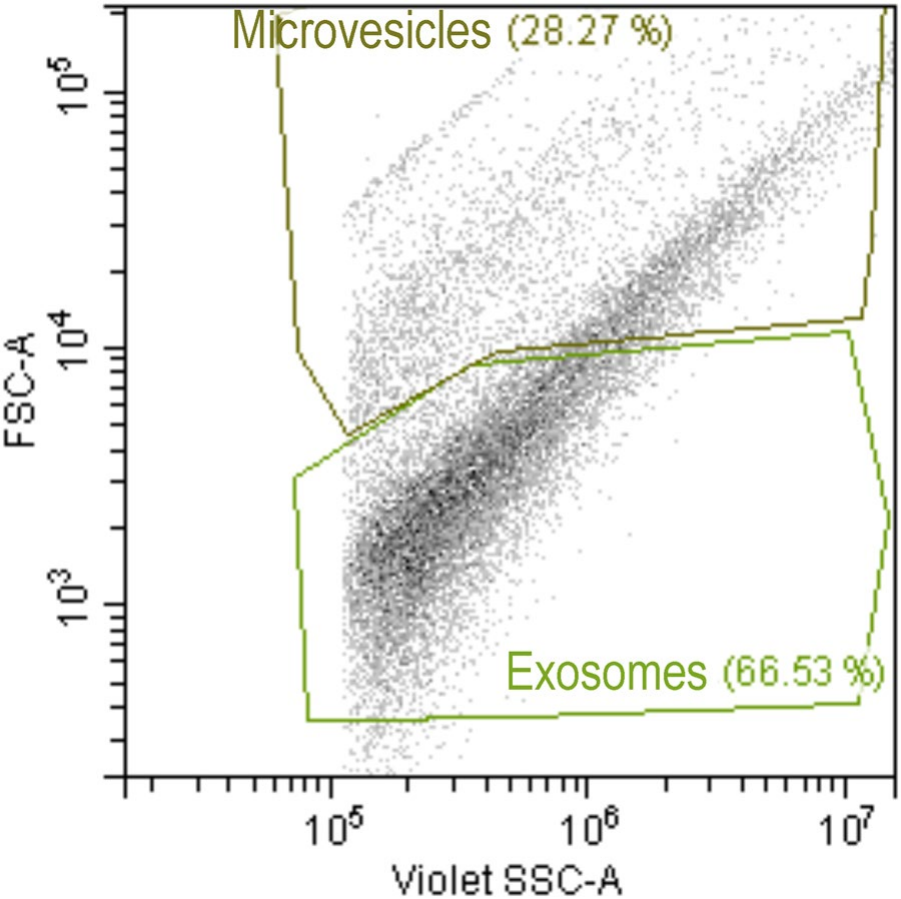
Discrimination of exosomes and microvesicles isolated from pig seminal plasma based on scatter parameters in CytoFLEX. Representative forward scatter (FSC-A, y-axis) vs side scatter (Violet-SSC-A, x-axis) dot plot of the two extracellular vesicles subtypes gated by size (exosomes [size: 50 to 120 nm] and microvesicles [size: 120 to 1000 nm]).

As a first step, the standard calibration kit containing fluorescent microspheres of known diameters confirmed that the CytoFLEX S flow cytometer was suitable for nanoparticle measurements. Its exceptionally good nanoparticle resolution and sensitivity allowed for accurate establishment of appropriate EV gates. The flow cytometric analysis was restricted to the EVs based on their characteristic properties in the Forward scatter (FSC) and Violet- Side Scatter (SSC). Two gates were established to detect EV subtypes based on their size properties as: exosomes, defined as nanoparticles with a diameter between 50 to 120 nm, and MVs, defined as nanoparticles with a diameter between 120 to 1,000 nm (Fig. 4). In order to distinguish true events from electronic noise and increase the specificity of EV detection, events in the EV gates were further discriminated by Carboxyfluorescein succinimidyl ester (CFSE)-labeling. The percentage of events positive to CFSE was 87.59 ± 1.27% [ranged from 80.45 to 94.35%]). The proportion of exosomes was higher (73.46 ± 0.56% [ranged from 70.60 to 77.32%]) than MVs (26.54 ± 0.56% [ranged from 22.68 to 29.40%]) in all EVs-preparations.

**Figure 5:**
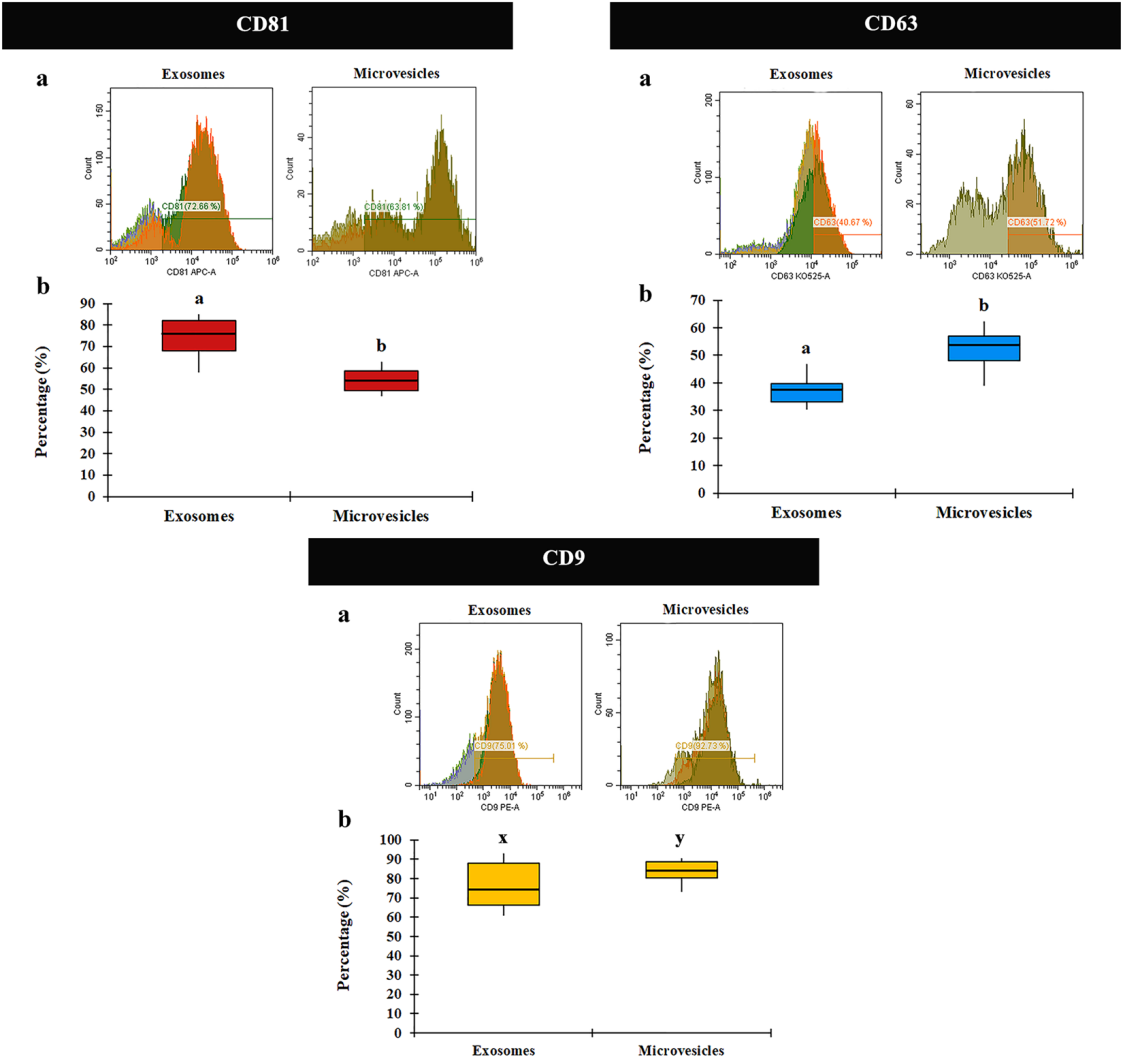
Flow cytometric analysis of tetraspanin CD81, CD63 and CD9 expression in extracellular vesicles (EVs) subtypes isolated from pig seminal plasma. (**a**) Histogram representative of CD81/CD63/CD9 expression in EV subtypes (exosomes and microvesicles). Fluorescence (CD81-APC-A, CD63-KO525-A and CD9-PE, x-axis), vs number of events (Count, y-axis). (**b**) Box-whisker plot showing variation in CD81/CD63/CD9 expression between EV subtypes of 12 ejaculates (one per boar). Boxes enclose the 25^th^ and 75^th^ percentiles; the line is the median; and the whiskers extend to the 5^th^ and 95^th^ percentiles. (**a**,**b**) and (x–y) indicate significant differences (P < 0.001 and P < 0.05, respectively) among the EV subtypes.

The results demonstrated that the three tetraspanins CD9, CD63 and CD81, were present in both EV subtypes in all examined samples of ejaculated seminal plasma. Moreover, each tetraspanin was differentially expressed depending on the EV subtype considered. The proportion of EVs expressing CD81 was higher (*P* < 0.001) in exosomes (74.23 ± 2.55% [ranged from 57.77 to 84.94%]) than in MVs (55.11 ± 1.56% [ranged from 46.81 to 62.65%]) (Fig. 5). In contrast, expression of CD63 and CD9 appeared lower in exosomes than in MVs (*P* < 0.001 and *P* < 0.05, respectively). The percentages of exosomes positive to CD63 and CD9 were 36.10 ± 2.08% (range: 17.78 to 46.79%) and 75.25 ± 3.24% (range: 60.77 to 93.17%), respectively. The percentage of MVs positive to CD63 and CD9 were 52.79 ± 1.77% (range: 39.00 to 62.31%) and 83.18 ± 1.57% (range: 73.01 to 90.52%), respectively (Fig. 5).

**Figure 6:**
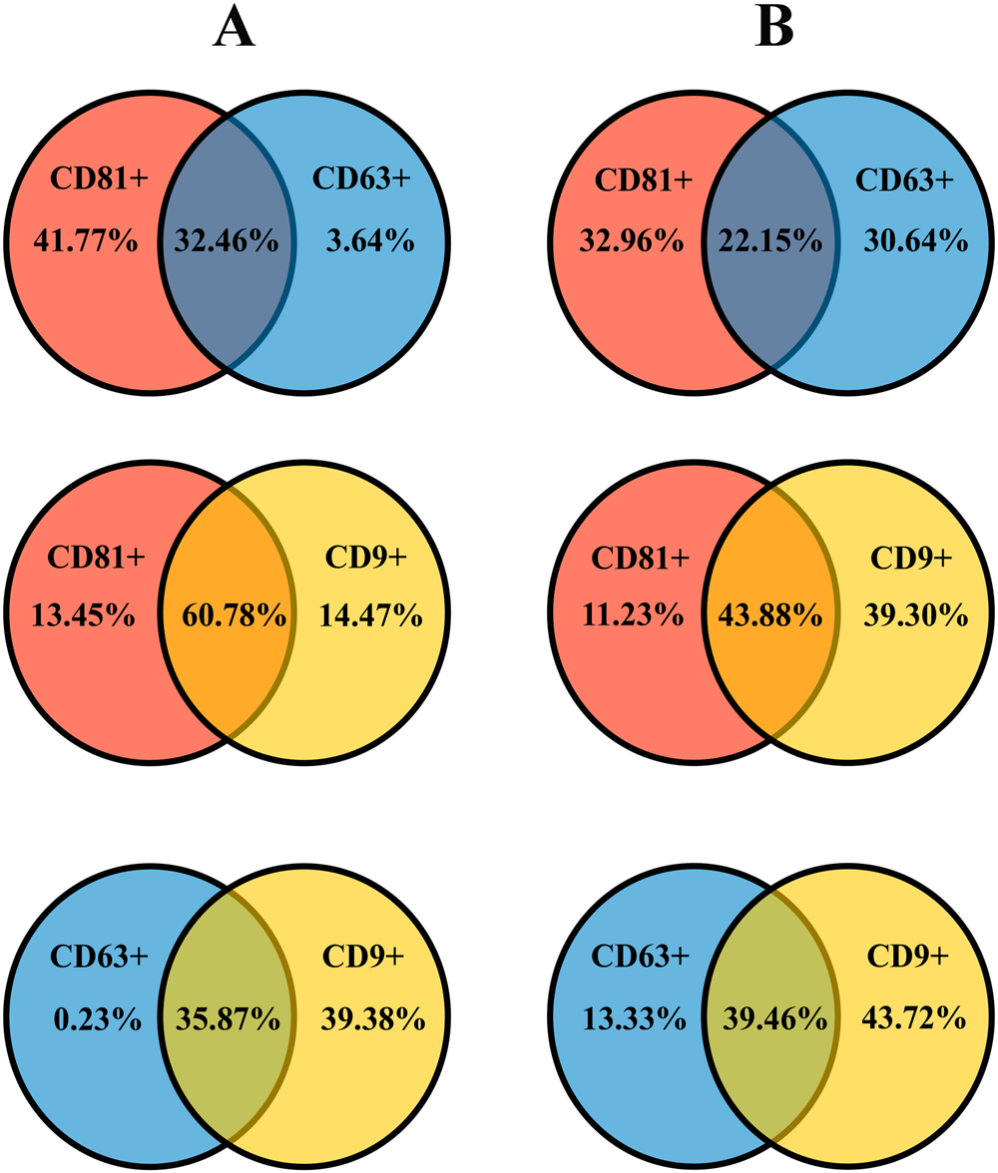
Venn diagrams showing the percentage of shared tetraspanins (CD9/CD63/CD81) in exosomes (**A**) or microvesicles (**B**). These extracellular vesicles, exosomes and microvesicles, were isolated from the pig seminal plasma and assessed by flow cytometry.

Simultaneous immunostaining allows dual immunofluorescent analysis by flow cytometry. Therefore, the co-expression of two tetraspanins was assessed. There were differences (*P* < 0.001) between the EV subtypes in the co-expression of tetraspanins CD63+ CD81+ and CD9+ CD81+ (Fig. 6). The proportion of CD63 + CD81 + was higher in exosomes (32.46 ± 1.81% [range: 15.73 to 40.91%]) than in MVs (22.15 ± 0.89% [range: 16.18 to 26.56%]). Likewise, the proportion of CD9 + CD81 + in exosomes was higher (60.78 ± 2.62% [range: 39.38 to 74.37%]) than in MVs (43.88 ± 1.92% [range: 32.70 to 52.98%]). However, the proportion of CD9 + CD63 + did not differ between exosomes (35.87 ± 2.05% [range: 18.17 to 46.32%]) and MVs (39.46 ± 1.21% [range: 34.03 to 48.13%].

## Discussion

Isolation of EVs from body fluids have been carried out with the use of different protocols^25^. However, none have proven precise enough to discriminate between EV subtypes in a single sample, which limits our understanding of the specific functions of each EV subtype^1^. In the absence of a standardized isolation technique, we used a modified ultracentrifugation protocol from the one considered the “gold standard” for EV isolation^26^. This procedure is based on several centrifugations, concluding with two serial ultracentrifugations. This sequential increase of the spinning force would aid best isolation of EVs. It was traditionally considered a useful procedure for selective isolation of exosomes^27^ based on the principle that MVs sedimented after spinning between 10,000 and 14,000 × g while that the smaller vesicles (exosomes) do it after spinning up to 100,000 × g. However, this procedure is not sensitive enough, and pellets recovered after ultracentrifugation contain mixtures of MVs and exosomes, as indicated previously by Bobrie *et al*.^28^ and Colombo *et al*.^1^ and confirmed in the present study. The electron microscopic images and DLS analysis revealed the heterogeneity in size of the EVs isolated after this serial ultracentrifugation. The electron microscopy also revealed differences in morphology and electron density among the isolated EVs, the presumable MVs having an electron-dense core while the smaller exosomes were electron-lucent. These results would agree with those previously recorded in semen samples from other mammalian species^29–31^ and support the notion that serial ultracentrifugation isolates a heterogeneous population of EVs. Seminal EVs have been classically classified according to their origin; as epididymosomes or epididymosome-like vesicles (from the epididymis) or as prostasomes or prostasome-like vesicles (from the prostate), with diameters ranging from 25 to 300 nm and from 30 to 500 nm, respectively^9^. Other studies simply used the term “exosomes” to identify any type of nanovesicle isolated by ultracentrifugation^11,15,32^. In short, there is no unified nomenclature to identify the presumptive different nanovesicles present in semen except for human, since samples from prostate secretion can be separately collected by transrectal digital massage^33^. Thus, unless a specific fluid is collected and processed, it is best to simply call them EVs^13^. Currently, the newest generation of flow cytometers allows accurate identification of nanoparticles and, therefore, it constitutes an excellent tool for evaluating EVs in a multiparameter way^19,34^. Our flow cytometry analysis confirmed, after labeling the EVs with the unspecific protein-dye CFSE, that the EVs isolated in pig semen were heterogeneous in size. Moreover, the efficient work of the cytometer facilitated a precise qualitative and quantitative analysis of the seminal plasma EVs, allowing to gate them by size into two well-defined EV subtypes which were, using the current EV nomenclature, considering exosomes when with a diameter below 120 nm and proportionally present in a greater number, against the less frequent MV, those with a diameter greater than 120 nm^17,35^.

Although the relationship between seminal plasma EVs and sperm epididymal maturation, motility, capacitation and acrosome reaction have been reported^9,13^, the specific role of these EVs on sperm functional stability is still controversial. For instance, while some studies indicated that seminal plasma EVs inhibit membrane reorganization during capacitation and the subsequent acrosome reaction^36,37^, others showed that they promote functional acrosome reaction^38,39^. The heterogeneity in size, morphology and perhaps in function of seminal plasma EVs, whose proportions could also vary between semen samples from different species, would explain these contradictory results. Therefore, it is mandatory to identify and isolate the different EV subtypes if we want to improve our knowledge of seminal plasma EVs function.

Antibodies against tetraspanins CD9, CD63 and CD81 have been useful for identifying EVs, but they are not considered able of selectively identifying the different subtypes of EVs, due to the overlapping presence of either type^7,13,28,40^. In agreement with the previous studies of Crescitelli *et al*.^40^ and Kowal *et al*.^7^, the cytometry study confirmed that both seminal plasma EV subtypes expressed CD9, CD63 and CD81. In addition, our results showed that the proportion of EVs expressing CD81 and CD9 was relatively high and that the expression of CD63 was relatively low in both exosomes and MVs. Such low proportion of EVs expressing CD63 is in agreement with Kowal *et al*.^7^, who demonstrated that CD63 was present only in a restricted EV subpopulation secreted from human dendritic cells. However, as a novelty, our results demonstrated that the proportion of EVs expressing the above tetraspanins differed between the two EV subtypes, which was previously suspected but never proven^1,23^. Welch *et al*.^41^ and Caballero *et al*.^42^ reported differences in tetraspanins expression in EVs isolated from human semen and bovine seminal fluid, respectively; yet they did not discriminate between the EV subtypes. In a comparative quantitative analysis of tetraspanin expression, we observed that the expression of tetraspanins CD63 and CD9 was higher in MVs than in exosomes while the expression of CD81 was higher in exosomes than in MVs.

The differences in tetraspanin expression would indicate that the exosomes and the MVs present in pig semen could have different functional activity. This statement is based on the concept that the function of EVs depends on their ability to interact with target cells, which is in turn determined by surface receptors existing in each EV subtype^43^. Tetraspanin complexes would be the EV surface receptors that would define the target cells to bind^21,22^. Caballero *et al*.^42^ demonstrated the relevance of tetraspanins in the functionality of EVs isolated from bovine seminal fluid, showing that EVs positive to CD9 transferred molecules to spermatozoa and that the transfer decreased when CD9 was blocked by specific antibodies. Additionally, these authors also evidenced that CD9 was associated with others tetraspanins, specifically with CD26. Similarly, the results of this study revealed an association of CD63 with CD9 and CD81 in exosomes, indicating a possible synergistic effect of both tetraspanins.

The specific functions of EVs are related to their cargo, endowed with proteins, lipids and genetic material, whose composition would dependent on the cell from they originated and would be responsible of modulating the functionality of target cells^13,43,44^. The tetraspanin expression profile of EVs probably varies according to their cargo. The study of Caballero *et al*.^42^ in EVs from bovine epididymal fluid provides some evidence supporting this hypothesis. They showed that the CD9 positive epididymosomes were particularly rich in proteins involved in sperm maturation and sperm-egg interaction. Similarly, Brzozowski *et al*.^45^ showed that the proteome of EVs isolated from human prostate cells differs by their degree of CD9-expression. Accordingly, our results could indicate that each seminal plasma EV subtype, with different tetraspanin profile, would have different cargo. It has been speculated that differences in EV subtype cargo could result in differences in biological function^35^. Probably, each seminal plasma EV subtype could play specific roles in fertilization process by transferring their contents through interaction with spermatozoa or the lining epithelium of the internal female genital tract. This, in turn, could condition pregnancy success. Accordingly, specific components of each EV subtypes could be used as biomarkers of infertility in the same way as it is intended to do for certain pathologies^35^. In addition, it has been demonstrated that supplementation of pig semen extenders with seminal plasma EVs improved sperm quality parameters^36^. Then, to identify the specific EV subtype and its cargo involved in this positive effect would allow the production of synthetic vesicles with the specific cargo for improving the effectiveness of semen extenders for artificial insemination (AI). Further studies are needed to make these possibilities viable.

In summary, the present study demonstrates that: (1) the serial ultracentrifugation of pig semen enables isolation of a heterogeneous population of EVs. (2) New generation flow cytometers are able to accurately identify such EVs and they allow to gate EVs in two size-different populations named exosomes and MVs. (3) Tetraspanins CD9, CD63 and CD81 are present in both EV subtypes, albeit exosomes and MVs depict different tetraspanin expression profiles. This different tetraspanin expression profile could indicate differences in cargo and cell target between exosomes and MVs. This study should be understood as a starting point to promote new trials aimed at separately isolating both EV subtypes and thereby elucidating possible differences in cargo and function. Fluorescence-activated cell sorters with increased SSC sensitivity, would be an excellent tool for achieving this goal.

## Methods

### Reagents and media

The chemicals used in the experiments were of analytical grade. All reagents, unless stated otherwise, were purchased from Sigma-Aldrich (St. Louis, MO, USA). The media were prepared under sterile conditions in a laminar flow hood (MicroH, Telstar, Terrasa, Spain). The basic media used to extend EVs and to dilute reagents and fluorochromes for flow cytometry was EDTA-free phosphate-buffered saline (PBS: NaCl 139 mM, KCl 2.7 mM, KH_2_PO_4_ 1.5 mM, Na_2_HPO_4_·7H_2_O 8.1 mM; with 0.058 g/L penicillin G and 0.05 g/L streptomycin sulphate; pH 7.1 ± 0.05; 287 ± 5 mOsmol/kg) 0.1 μm filtered (0.1 μm filtered-PBS; Millex® syringe filter units, Merck, Darmstadt, Germany).

### Animals and ejaculates

All procedures that involved animals were performed according to international guidelines (Directive 2010-63-EU) and approved by the Bioethics Committee of Murcia University (research code: 639/2012).

Single ejaculates (volume = 401.25 ± 73.01 mL [ranged from 261 to 486 mL]) were collected from 12 healthy, mature (aged 18–36 months) and fertile breeding boars (one ejaculate per boar) using a semiautomatic collection method (Collectis®; IMV Technologies, L’Aigle, France). The boars were housed in an AI centre (AIM Ibérica, Topigs Norsvin España, Calasparra, Murcia, Spain). Ejaculates fulfilled the standards of quantity and sperm quality thresholds for the preparation of semen AI-doses (more than 200 × 10^6^ spermatozoa/mL, 70% motile spermatozoa, and 75% of morphologically normal spermatozoa).

### Isolation of EVs

The EVs were isolated from seminal plasma by serial ultracentrifugation following a modification of the procedure described by Thery *et al*.^46^. First, the ejaculates were centrifuged twice (1,500 × g for 10 min at room temperature [RT]; Rotofix 32 A; Hettich Centrifuge UK, Newport Pagnell, Buckinghamshire, England, UK) immediately after collection to obtain the bulk seminal plasma. Aliquots (5 mL) of seminal plasma were stored in cryotubes and sent in insulated containers with dry ice to the Andrology Laboratory of the Veterinary Teaching Hospital of the University of Murcia (Spain). At the laboratory, the seminal plasma samples were stored at -80 °C (Ultra Low Freezer; Haier Inc., Qingdao, China) for two weeks. After this storage period, the seminal plasma samples were thawed at RT inside a dark chamber and centrifuged at 10,000 × g for 30 min at RT to remove any possible remaining sperm- or other cell debris. The supernatants (2.5 mL) were centrifuged at 100,000 × *g* for 70 min at 4 °C using a swinging-Bucket rotor type SW41 Ti on a Beckman Optima L-100XP ultracentrifuge (Beckman Coulter, Brea, CA, USA). The pellets were then washed with 0.1 μm filtered-PBS and centrifuged at 100,000 × *g* for 70 min at 4 °C. The resulting pellets were resuspended in 0.1 μm filtered-PBS (50 μL) and stored at -80 °C in 5-μL aliquots (EVs-preparations) until analysis.

### Assessment of EVs morphology by transmission electron microscopy

A modification of the procedure described by Thery *et al*.^46^ was used. Briefly, EVs-preparations (5 μL, n = 12) were fixed in 2% paraformaldehyde (PFA) and placed onto formvar carbon coated electron microscopy grids. After incubation for 20 min at RT in a dry environment, the EVs-preparations were fixed in 1% glutaraldehyde for 5 min, washed in distilled water (8 water washes [2 min each one]), contrasted in 2% uranyl-oxalate for 5 min and embedded in a mixture of 4% uranyl-acetate and 2% methyl cellulose for 10 min on ice. The EVs were examined and photographed using JEOL JEM 1011 TEM (JEOL Ltd., Tokyo, Japan) with an Orius SC200 camera (Gatan, Evry, France).

### Assessment of EVs morphology by scanning electron microscopy

The EVs-preparations (5 μL, n = 12) were processed for SEM following the procedure described by Wu *et al*.^47^. The EVs-preparations were resuspended in 0.1 μm filtered-PBS (up to 0.2 mL), fixed in 2% PFA and diluted in distilled water in serial dilutions (up to 1/32). To make the surface conductive, a coating of 2–5 nm gold-palladium was applied by sputtering before imaging by SEM Apreo S™ (Thermo Fisher Scientific, Waltham, Massachusetts, USA).

### Assessment of EVs size distribution by dynamic light scattering

The size distribution of vesicles present in EV-preparations was measured by DLS. Briefly, before to DLS measurements, the EVs-preparations (5 μL, n = 12) were extended with 0.1 μm filtered-PBS (45 μL) and shaked to disaggregate possible clumps of EVs. Then, 50 μL of each sample were added to a cuvette with a 10 mm pathlength. The analysis was conducted using a Nano Zetasizer (Malvern Instruments, Malvern, UK) at RT, operating at 633 nm and recording the back scattered light at an angle of 173°. The light scattering was recorded for 150 s, with 3 replicate measurements for each sample. Dispersion Technology Software v.5.10 (Malvern Instruments) was used to transform DLS signal intensity to size distribution. The peak maximum of the gaussian function was used to estimate EV diameter (nm). The intensity-based distribution was re-calculated to a volume. The results were expressed as volume and intensity size distribution.

### Evaluation of tetraspanin EV-expression by high-resolution flow cytometry

The EVs-preparations (5 μL, n = 12) were incubated with anti-CD9-phycoerythrin (BIO-RAD, California, USA), anti-CD63-Alexa Fluor® 405 (Santa Cruz, Heidelberg, Germany), anti-CD81-allophycocyanin (BD Bioscience, Franklin Lakes, NJ, USA) and CellTrace ™ CFSE (Thermo Fisher Scientific) for 30 min at RT. Following incubation, samples were resuspended in 0.1 μm filtered-PBS to a final volume of 200 μL. Data were acquired and analyzed using a CytoFLEX S high-resolution flow cytometer (Beckman Coulter, Life Sciences Division Headquarters, Indianapolis, USA), equipped with red (638 nm), blue (488 nm), yellow (561 nm) and violet (405 nm) lasers. The distillated water (0.1 μm filtered) was used as sheath fluid. The flow cytometer performance was verified using a standard calibration kit (Nanobead Calibration Kit, Bang Laboratories Inc., Technologies Drive Fisher, Indiana, USA), composed by microspheres with diameters of 50 and 100 nm with an internalized fluorescent dye. These beads were used to set the EV gate and to calculate the EV counts. Optic configuration was set to use Side Scatter information from 405 nm laser (Violet-SSC-A). FSC and Violet-SSC-A were set in a logarithmic scale, and the fluorescence channels were set at logarithmic gain. The 0.1 µm-filtered PBS was analyzed prior to sample acquisition to ensure removal of background noise. The analysis was restricted to the EVs based on characteristic properties of these vesicles in the FSC (size) and Violet-SSC-A (complexity). Each sample was analyzed using the low flow rate setting (10 µL/min). At least 10 × 10^3^ events were acquired for analysis.

### Statistical analysis

Data from the flow cytometric analysis were analyzed using IBM SPSS Statistics 19.0 (IBM, Armonk, NY, USA). Residual data for each statistical variable were evaluated using the Kolmogorov-Smirnov test to check the assumption of normality, and data that were not normally distributed were arcsine transformed. One-way ANOVAs were performed to investigate differences on tetraspanin expression between the EV subtypes. Statistical significance was defined as *P < *0.05. Data are presented as the means ± SEM.

## Data Availability

The data used and analyzed during the current study are available from the corresponding author on reasonable request.
